# Enabling Medicine Reuse Using a Digital Time Temperature Humidity Sensor in an Internet of Pharmaceutical Things Concept

**DOI:** 10.3390/s20113080

**Published:** 2020-05-29

**Authors:** Terence K. L. Hui, Parastou Donyai, Rachel McCrindle, R. Simon Sherratt

**Affiliations:** 1 Department of Biomedical Engineering, School of Biological Sciences, University of Reading, Reading RG6 6AY, UK; t.hui@reading.ac.uk (T.K.L.H.); r.j.mccrindle@reading.ac.uk (R.M.); 2 School of Pharmacy, University of Reading, Reading RG6 6AY, UK; p.donyai@reading.ac.uk

**Keywords:** medicine reuse, reduce medicinal waste, intelligent pharmaceutical packaging, medicine re-dispensing technologies, TTI (time temperature indicator), internet of pharmaceutical things, IoT

## Abstract

Medicinal waste due to improper handling of unwanted medicines creates health and environmental risks. However, the re-dispensing of unused prescribed medicines from patients seems to be accepted by stakeholders when quality and safety requirements are met. Reusing dispensed medicines may help reduce waste, but a comprehensive validation method is not generally available. The design of a novel digital time temperature and humidity indicator based on an Internet of Pharmaceutical Things concept is proposed to facilitate the validation, and a prototype is presented using smart sensors with cloud connectivity acting as the key technology for verifying and enabling the reuse of returned medicines. Deficiency of existing technologies is evaluated based on the results of this development, and recommendations for future research are suggested.

## 1. Introduction

An estimated GBP 300 million worth of prescribed medicines are wasted ever year in the UK alone, and similar situations are reported in other countries [1,2]. Medication non-adherence and many other causes, such as over prescribing, over ordering, change of treatment, or a patient dying, create unused and unexpired prescribed medicines which cannot be returned to pharmacies for re-dispensing due to government policies [3]. The indirect financial impact of medicinal waste is as important as the financial costs because of public health risks and environmental pollution from the disposal of these medicines. Medicine reuse seems to be a partial solution and it proves to work when legal restrictions are resolved in certain areas of the world such as the USA and Greece [4]. This is the idea that medicines dispensed to one patient, if unused and returned to a pharmacy, can be re-dispensed for another patient. However, even when the re-dispensing of returned medicines is not prohibited by local laws and regulations, there are further obstacles blocking the effective implementation of a medicine reuse programme.

Previous qualitative studies reveal that stakeholders in the pharmaceutical sector generally accept the concept of medicine reuse if certain quality and safety requirements are met [5,6,7]. The activities of returning and reusing prescribed medicines are human behaviours, and Alhamad et al. [8] reported similar requirements that could influence stakeholders’ beliefs based on the Theory of Planned Behaviour (TPB). A comprehensive and structured review of studies in the area of medicine reuse, summarises and translates the requirements identified into a proposed Reuse of Medicines through Informatics, Networks and Digital Sensors (ReMINDS) ecosystem, where technologies could enhance pharmaceutical packaging to facilitate the reuse of medicines and reduction of medicinal waste [9]. Applying Internet of Things (IoT) technologies to pharmaceutical packaging could be the key enabler shaping stakeholders’ behaviours through perceived control and behavioural beliefs.

The use of technology to reassure stakeholders of safety and quality is not new. Technologies have long been applied to food packaging for monitoring the freshness of packaged food, and sensors are frequently embedded inside the packaging/facilities of where food is stored [10]. Monitoring of the storage conditions as well as facilitating ‘track and trace’ in supply chain management are the major foci in pharmaceutical packaging technologies [11]. However, technology to enable reuse of medicines is not common and the corresponding research is not at all mainstream [9].

Derived from the proposed ReMINDS ecosystem, a novel Internet of Pharmaceutical Things (IoPT) concept could seamlessly enlist medicines to the IoT platform through technology embedded pharmaceutical packaging [9]. Medicines (packaging, bottles, even individual tablets/capsules) become Things connecting through the IoPT to all stakeholders facilitating medicine reuse. At the same time, up-to-date patient information may also be shared through this platform to authorised persons for tele-healthcare. Quality and safety requirements can then be met using suitable sensing and connectivity technologies applied to the pharmaceutical packaging. A self-monitoring analysis and reporting technology (smart) [12] sensor based on the IoPT concept would allow patients to decide whether the medicines can be returned to pharmacies or not, enable pharmacists to validate returned medicines for re-dispensing, empower pharmaceutical companies to track and trace the products through sustainable pharmaceutical supply chain management, provide data for medical professionals to investigate therapeutic effectiveness, and create opportunities for governments and the general public to change and monitor national health policies.

This article presents a novel IoPT concept as an extension of the IoT architecture facilitating medicine reuse, and an application of the concept to develop a smart sensor is discussed. A Digital Time Temperature Humidity Indicator (dTTHI), acting as an IoPT smart sensor, has been researched and constructed according to the requirements derived from the IoPT concept. Commercial off-the-shelf components were chosen to evaluate the readiness of existing technologies for IoPT, and recommendations were made for future research on any missing functions and components. Section 2 illustrates the design criteria for building the IoPT architecture for medicine reuse, and major requirements are recommended as guidelines for developing the basic building blocks. Details of hardware and firmware implementation complying with the proposed design requirements are depicted in Section 3. The choice of hardware components that enables the sensing and connectivity are discussed, and the software algorithm facilitating medicine reuse is presented. Section 4 discusses the pros and cons of available technologies for implementing the IoPT smart sensor, and improvements for future research are also reviewed. Finally, conclusions and future works are summarised in Section 5.

## 2. Methods

The ReMINDS ecosystem proposed by the same authors of this paper provides the background for building a technological framework for enhancing pharmaceutical packaging to facilitate medicine reuse [9]. A seamless connection between stakeholders in the pharmaceutical sector exchanging real-time information is the key to sustaining the proposed ecosystem, and IoT technologies can empower the establishment of this information platform. Enlisting medicines as a connected IoT device, or Thing, with specific functions is the major factor differentiating IoPT from a traditional IoT concept. This section describes the necessary functions for establishing a ReMINDS architecture, and makes recommendations on designing and building the ecosystem and its components.

According to previous qualitative research [5,6,7,8,9], the major requirements for reusing dispensed medicines can be classified into three categories: quality, safety and others (see Table 1 for details). Applying IoT technologies to pharmaceutical packaging may be a solution to facilitate the concept of medicine reuse [9]. Pharmaceutical packaging is regarded as the “key facilitator” for building a patient-medication relationship [13], it not only protects packaged medicines, but also establishes an effective interface with all stakeholders through information exchange. IoT technologies help record and share complete sensor data related to the environment of packaged medicines after leaving pharmacies. smart sensors based on the IoT concept can be enhanced to include medicines and all stakeholders creating the IoPT ecosystem for medicine reuse. *tti are popular quality indicators for food packaging reporting the freshness of packaged food based on visual illustration of time-temperature history [14], but their lack of a digital interface and connectivity to the Internet restricts usage in smart packaging [15]. The design of a dTTHI, similar to a *tti in functionality, is reported in this paper as an example illustrating the application of the IoPT concept to a smart sensor platform supporting the validation of returned medicines ready for re-dispensing.

### 2.1. Design Criterion

**Figure 1:**
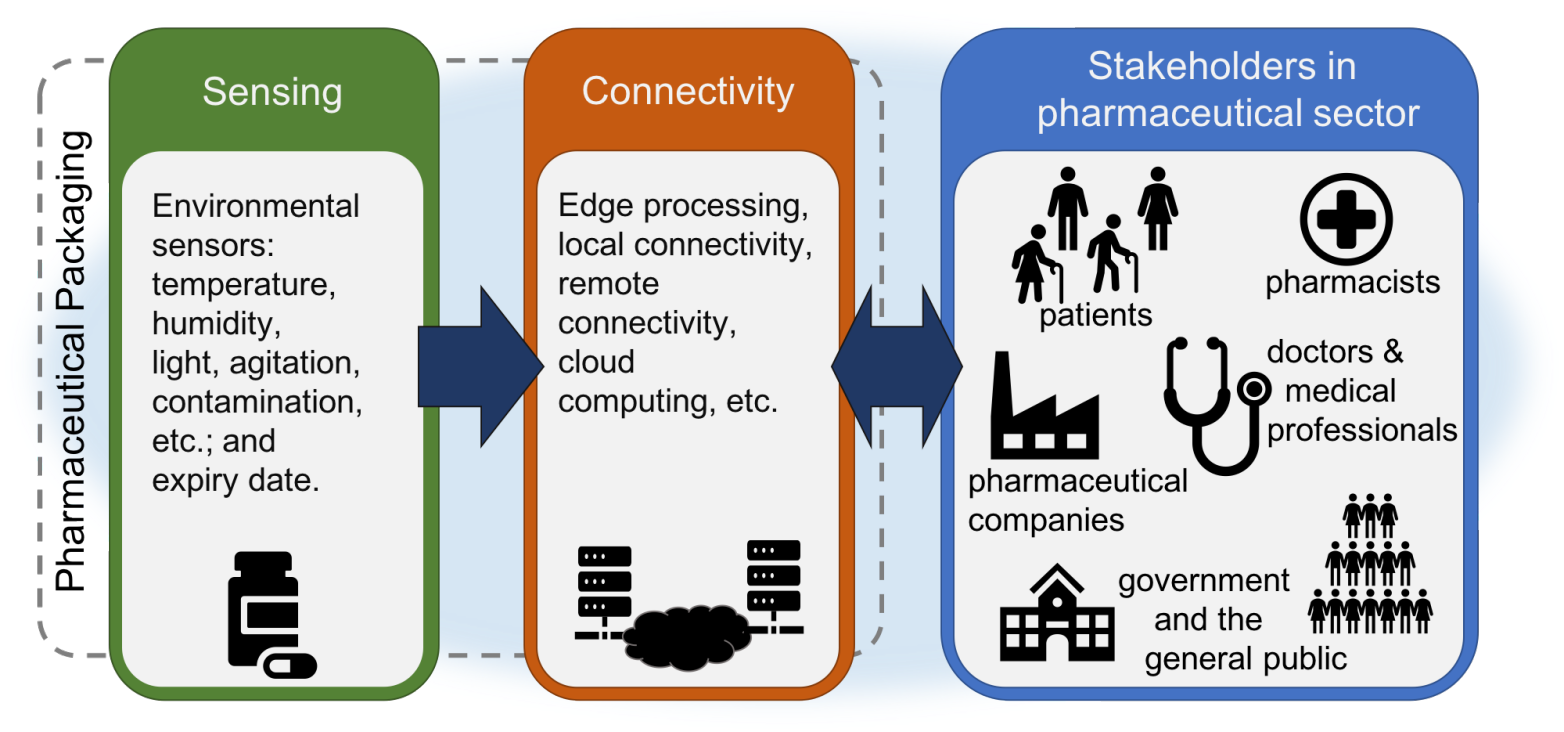
Typical IoPT architecture for medicine reuse derived from the ReMINDS ecosystem [9].

From a technology perspective, a complete IoPT ecosystem for reusing dispensed medicines may fundamentally consist of a smart sensor platform connected to the Internet. Raw sensor data is recorded and uploaded to a cloud server sharing information to individual stakeholders according to their specific requirements. Figure 1 illustrates a typical architecture of a proposed IoPT for medicine reuse. Local sensing and stakeholder connectivity are the core components for the smart sensor platform. Its size and power consumption may not be the major considerations for the stakeholders in the pharmaceutical sector, but they are critical factors for a successful implementation of technologies in the limited space available within or on pharmaceutical packaging. The costing of such an implementation is a complex issue which is affected by technologies, component costs, design strategy and the way to achieve economies of scale for mass deployment.

Sensing of parameters within the environment is the first step in monitoring pharmaceutical packaging storage conditions in order to resolve the quality concerns in medicine reuse. According to Table 1, the major environmental parameters affecting the quality of returned medicines are:

(i) Temperature
(ii) Relative humidity
(iii) Light intensity
(vi) Agitation
(v) Contamination

Parameters (i–iii) apply to package surroundings where common environmental sensors based on traditional or thin film technologies are required to take measurements. Thin film printed electronics may be a better choice for seamless integration with pharmaceutical packaging, such as flexible hybrid electronics or nanotechnology [16,17]. However, traditional fixed silicon substrate methodologies provide ample selections of sensor components with smaller size, lower power and better functionality. Parameters (iv)–(v) are more concerned with the quality of medicines themselves, where physical or mechanical sensors monitor unexpected motion or agitation [18], and chemical or biological sensors characterise contamination to packaged medicines [19]. Expiry date monitoring is also crucial since it overrides the results obtained from environmental sensing when packaged medicines have come to the end of their period of validity. Continuous comparison of the current date with expiry date triggers alerts to patients and relevant stakeholders to take necessary actions, such as making decisions about reusing or incinerating unused returned medicines. A dynamic editable expiry date may be an enhancement to the fixed date hard-coded at point of manufacture in pharmaceutical companies thereby allowing a shortening (or extension, if applicable) of the expiry date when certain environmental parameters exceed (or fall within) predefined limits [20]. During state emergencies or other events that cause a shortage of medicines, perhaps the pharmaceutical companies could remotely extend the expiry date by relaxing the validation standards.

Technologies applicable to pharmaceutical packaging that comply with safety requirements are not common [9]. Static temper-proof technologies for indicating seal-broken packages, based on visual inspection, have been implemented on medicine and food packaging for a long time [21]. Digital interfaces for tamper monitoring may require a detection of changing impedance of a close-loop circuit, such as breaking a conducting wire or lowering the conductance of a contact through opening a package or removing a capsule in a blister pack. A sensor connecting to a Microcontroller (MCU) through a General Purpose Input/Output (GPIO) or Analog-to-Digital Converter (ADC) port is required. Counterfeit protection may be more effectively implemented through integrating with cloud computing since a central database registering the details of each medicine along the complete pharmaceutical supply chain from manufacturers to patients may provide a better track and trace record for individual medicines. Details for each medicine include its unique identification, the production data, transportation tracking reports, dispensing records, storage and consumption conditions, etc. An unalterable, distributed ledger for transactions based on the block chain technology, for example, can put security and privacy into anti-counterfeit consideration when proper cybersecurity is implemented. Internet connectivity together with cloud computing can be a basic architecture for the ReMINDS ecosystem facilitating tamper-proofing and anti-counterfeiting of packaged medicines.

Other requirements listed in Table 1 refer to concerns coming from specific stakeholders and may not be resolved using technologies alone. Incentives for returning and re-dispensing unused prescribed medicines create extra workload for stakeholders especially patients and pharmacists. An incentive scheme such as gaining financial or non-financial credits according to the reuse quantity may motivate positive actions towards reusing medicines, and this can be implemented through cloud database services registering individual contributions. However, security and privacy must be implemented to the scheme in order to keep personal information hidden so that the whole process can be conducted anonymously. Cost of implementation covers both the costs incurred from putting technologies in pharmaceutical packaging, and installing an IoPT infrastructure. The latter can be shared by relevant stakeholders or governments and is usually a one-off expenditure, however, the additional cost for turning traditional packaging into smart or intelligent packaging may be a difficult problem to be solved. Lowering the cost of additional components embedded in pharmaceutical packaging may be achieved through: (a) integrating technologies into packaging such as printing digital electronics onto packaging materials, and/or (b) customising basic functions into an application specific integrated circuit (ASIC) to get the best cost performance ratio, and/or (c) recycling technology embedded packaging materials for different types of medicines through reconfigurable design, and/or (d) expanding usage through mass deployment to achieve economy of scale. Legal issues may require changes in local law and regulations, professional standards, and standard operating procedures which could be supported by scientific evidence and tools such as comprehensive quality and safety validation methodologies. Social norms are powerful ways to motivate human behaviours towards common goals complying to standards and norms of a group [22], and information technology such as social networking could help facilitate the goal of medicine reuse through implementing a ReMINDS social media cloud service [23]. On-site and off-site collection and distribution could be part of a modern pharmaceutical supply chain management where real-time information for all related activities is recorded and uploaded to a shared cloud storage. Again, connectivity and cloud computing based on IoT technologies linking every pharmaceutical stakeholder in the ReMINDS ecosystem is necessary to fulfil the list of “other requirements”.

Connectivity plays a critical role in both the IoT and the IoPT concept, and it helps upgrade a digital sensor to a smart sensor. Local connectivity provides data sharing of sensing results through zero to short distance communication mainly to patients and pharmacists, and remote connectivity extends information sharing to every stakeholder in the whole medicine reuse ecosystem [9]. A dynamic changing single or multiple colour indicator (e.g., a Light Emitting Diode (LED)) or a graphical display module (e.g., a Liquid Crystal Display (LCD) or e-paper display) driven by a MCU can display the sensing results at zero distance from the packaging surface. When a tangible interface is not available, a short range radio device such as Radio Frequency Identification (RFID), Bluetooth (BT) or WiFi enables the sensor data to be transferred to a receiver at a close distance from the pharmaceutical packaging (e.g., using a smartphone, or laptop computer, etc.). Edge computing combined with local connectivity allows sensor data to be shared to patients and pharmacists to take immediate actions usually based on a summary of the sensor results. Actions to be taken include but are not limited to validation of which medicines are suitable for returning to community pharmacies. Remote connectivity enlists cloud computing to make the IoPT complete. Individual stakeholders in the ecosystem may require different information derived from the raw sensing data collected from smart sensors on pharmaceutical packaging, for example, pharmacists may utilise a complete profile of environmental sensing data to make final judgements on re-dispensing returned medicines, pharmaceutical companies may utilise variations of sensing results to shorten or extend the expiry date, doctors and medical professionals may utilise sensing results to perform therapeutic analysis on medication adherence, government agencies may utilise the overall profile of returning and re-dispensing dispensed prescribed medicines to monitor the effectiveness of medicine reuse, etc. All this information can be retrieved from the raw data based on data analytics provided by private or commercial cloud services. Due to security and privacy issues, the general public may only be able to extract filtered data enabling individuals to participate in influencing government policies and gain public benefits. Remote connectivity can be established through a gateway routing local data to a cloud server on the Internet, or via a direct Internet connection (through base stations) using a long range Low Power Wide-Area Network (LPWAN) such as Long Range (LoRa), Sigfox or contemporary cellular radio for IoT (e.g., Narrowband Internet of Things (NB-IoT), Long Term Evolution for Machines (LTE-M), etc.). An ecosystem from a technology perspective for medicine reuse is thereby established through this IoPT concept where medicines are connected to all related stakeholders including patients, pharmacists, medical professionals, pharmaceutical companies, governments, as well as the general public through the Internet.

Size and power consumption are technical considerations for implementing smart IoPT sensors, and they are common constraints for designing resource limited IoT edge devices such as sensor nodes [24]. Shrinking dimensions of silicon electronics and boosting of their energy efficiency have never stopped since the demands from industry for more processing power (e.g., supercomputers, neural networks, etc.) and smaller form factors (e.g., IoT sensor nodes, implantable biosensors, etc.) have never ended [25]. Moore’s law has seemed to be slowing down in recent years but 3-D stacking, nanotechnology, and innovative processing and power management may help to keep pushing the energy efficiency of digital electronics in a forward direction [26]. As inspired by Koomey and Naffziger [27], a ubiquitous, cheap, small footprint, and ultra-low power MCU architecture provides a solution for building a long battery life IoPT smart sensor by enabling each function or module in a complete system to have its own processing power and to only be active when necessary. An extremely low power sensor module operating in microampere or even nanoampere current performs regular sensing on its own and triggers the main MCU to work when a threshold is reached. Together with a wireless System on Chip (SoC) which is in deep sleep mode most of the time and is active once a day to upload data to the cloud, a complete smart IoPT sensor is built. Thin film printed electronics may help further shrink the overall size of smart sensors but this technology is still not mature enough for immediate implementation [9].

### 2.2. Design Requirement Recommendations

IoPT utilises existing IoT technologies to establish a technology architecture providing a platform connecting medicines as Things to the Internet. IoPT is not only an extension of IoT, but also a collection of tools and concepts establishing a complete system facilitating the goal of medicine reuse. According to the design criteria discussed above, the following design requirements provide guidelines for building the IoPT system and its individual components.

(1) Environmental Parameter Sensing: Related to the monitoring and recording of environmental parameters regarding package surroundings, the sensing range, accuracy and sampling frequency are major concerns.
(2) Expiry Date Monitoring and Management: Related to the functions of setting, resetting and alerting regarding a dynamic expiry date system.
(3) Safety Protection: Related to the algorithms protecting medicines from tampering and counterfeiting.
(4) Internet Connectivity: Related to the ways connecting to the Internet wirelessly.
(5) Cloud Computing: Related to the web services empowering various data analytics for sharing specific information to stakeholders.
(6) Size and Power Constraints: Related to the limitations concerning product sizes and power consumptions of technology embedded smart sensors.
(7) Usability: Related to the user interfaces aiming for easy to use and easy to learn ways of validating unused medicines.
(8) Cost Effectiveness: Related to the effective cost of implementing the whole IoPT architecture and its components.
(9) Incentive Facilitation: Related to the administration of stakeholders’ incentives for motivating positive behaviours for reusing medicines.
(10) Social Norms Support: Related to the setup of digital social norms shaping stakeholders’ behaviours normative beliefs.
(11) Security and Privacy Protection: Related to the protection against unauthorised access to all data of the prescribed medicines through provision of end to end protection.

Some of the requirements are targeted to be implemented in the pharmaceutical packaging when local processing is required, but tasks requiring higher computing power and/or involving external resources may be done remotely through cloud computing. An example smart sensor, the dTTHI, was developed according to the above design requirements to illustrate the application of the recommended design requirements to establish the IoPT infrastructure and its components.

## 3. Results

Translated from the design criterion discussed in the last section, the fundamental components for designing an IoPT smart sensor platform facilitating medicine reuse include:

ⓐ A low power environmental sensor that performs continuous sensing and triggering functions without an external controller to save energy.
ⓑ A main MCU which infrequently reads sensor data and uploads results to the Internet for cloud computing, it also updates the on-packaging display for zero distance local connectivity.
ⓒ A wireless module which provides Internet connectivity through a wireless local area network.
ⓓ An ultra-low power graphical display module that indicates the current sensing results as well as the quality status of the packaged medicines.
ⓔ An energy supply based on a lithium battery that maintains the operations of the smart sensor.
ⓕ A cloud computing platform that stores sensing data collected from the smart sensor and allows sharing of information to all stakeholders through various web services.
ⓖ Computing devices (e.g., desktop computers, laptop computers, smartphones, etc.) that connect to cloud servers and retrieve bespoken information from anywhere with Internet connectivity.

**Figure 2:**
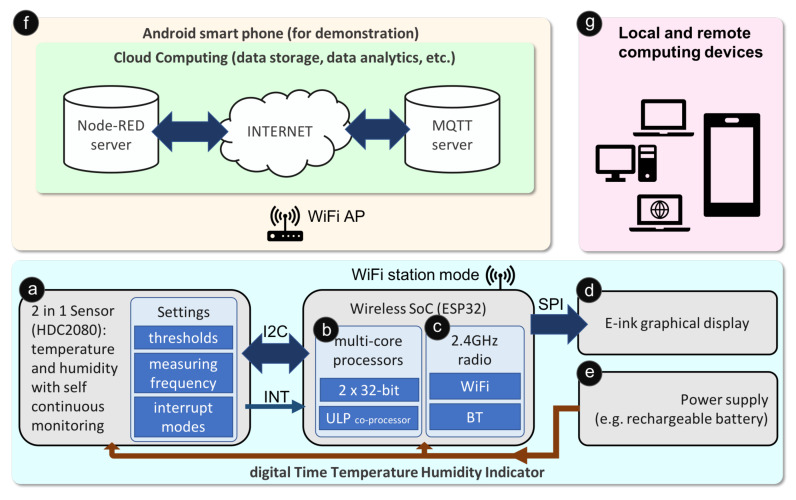
Block diagram of a dTTHI for medicine reuse based on the IoPT concept.

Figure 2 depicts a block diagram of a typical IoPT smart sensor platform, with the implementation of each function being described in detail below.

Sensors are the core components that track environmental changes, with selection of sensors being a straight forward process with physical size, power consumption and digital interface as the main considerations for each type of sensing. For demonstration purposes, the dTTHI only incorporates temperature and humidity sensors for this current study. Additional sensors for sensing other environmental parameters could however follow the same algorithm simply by extending the connection to the additional sensor interfaces commonly through a serial communication protocol such as Inter-Integrated Circuit (I2C) or ADC. As shown in Figure 2 block ⓐ, HDC2080 [28] was chosen as the environmental sensor. This is a 2-in-1 module combining temperature and humidity sensing on a single chip. The main reasons for selecting HDC2080 were: (i) its self continuous sensing function where the sensor module can perform automatic temperature and/or humidity measurements at a frequency set by the main MCU, (ii) its extremely low operating power during idle (50 nA) and full function (500 nA) modes, and (iii) its internal threshold triggering function which can trigger a hardware interrupt to the main MCU once a preset threshold setting is reached, and with these thresholds able to be adjusted dynamically by the main MCU through programming. Therefore, an ultra-low power sensing was achieved since most of the time the main MCU and the wireless frontend were in deep sleep mode, with the only power drain coming from the sensor standby current plus the measuring mode current at a preset frequency. One measurement per minute was set for regular temperature and humidity monitoring; thus, the average power consumption for the whole sensor was 1.65uW when there was no activation triggered by the threshold comparison logic.

A low-power, inexpensive and multi-core wireless SoC was selected as the main MCU (block ⓑ) and the wireless connectivity (block ⓒ) for the dTTHI. In order to save energy, both the MCU and the radio were switched dynamically between normal and deep sleep modes through programming [29]. The two 32-bit CPU cores act as the main controller for the whole dTTHI device, including interfacing with the HDC2080 sensor module through I2C, driving the graphical display using Serial Peripheral Interface (SPI), and connecting to cloud servers based on the on-chip radio. ESP32 provides both WiFi and BT baseband sharing a single 2.4GHz radio frontend, thus, only one wireless protocol is active at a single time. The present example uses WiFi protocol only and is set to a station mode to connect wirelessly to an Access Point (AP). An Ultra Low Power (ULP) coprocessor embedded in ESP32 could be used as an alternative power saving solution to read the sensor regularly during ULP mode and to wake up the main MCU at the timer interrupt (here, the timer was set to once per day) or when the readings exceed the thresholds, however, the standby power of the ULP was higher than HDC2080’s self measurement mode so it was not used in the current design. In order to achieve minimum power consumption, all external components supporting the ESP32, such as the voltage converter and the battery charging circuits, must all be configured to consume minimum possible energy.

An e-ink display (block ⓓ) was selected to provide a zero distance local connectivity especially to patients for conveniently checking medicine quality. E-ink technology was chosen due to its zero-power sustaining feature, its relatively high resolution that can show a better graphical image, and its maturity for easy purchase. A 2.13 inch e-ink display module [30] supporting a partial update function was used in the dTTHI in order to keep the refreshing cycles short, with the content updated once every day when the main MCU woke up during cloud server connection.

Size constraint and the requirement for high peak current during WiFi transmission were the two factors for choosing the type of power supply. A 3.7V 110mAh lithium rechargeable battery with dimensions 35 × 15 × 2mm was used to supply the power ⓔ to the whole dTTHI device.

Cloud computing (block ⓕ) plays a major role in the whole IoPT ecosystem for medicine reuse by connecting medicines with all stakeholders. In the current example, two cloud servers were established to support information sharing between stakeholders: a MQ Telemetry Transport (MQTT) server for exchanging short messages between smart sensors such as the dTTHI in the current study, and a Node-RED server for performing information management and other administration functions such as analysing the raw data or displaying infographics. A WiFi router was necessary in the whole platform routing the WiFi signals between the dTTHI and the Internet cloud servers. For demonstration purposes, an Android smartphone (a Google Pixel model running Android OS version 10) was used to simulate a cloud computing platform. Using a terminal application (Termux, https://termux.com), an MQTT broker application (mosquitto version 1.5.7, https://mosquitto.org), and a Node-RED server (node-red v0.20.3, https://nodered.org) were installed on the smartphone. The Android smartphone also acted as a WiFi AP using tethering, thereby, setting up its own network. Finally, the smartphone simulated a cloud computing platform that could be connected directly through a gateway on its own.

Any computing devices such as notebook/laptop computers, desktop computers, tablets or smartphones (block ⓖ) could connect to the cloud computing platform and access the raw sensor data or processed data from the servers. These computing devices could join the network in the present IoPT example through the AP created by the Android smartphone or they could connect remotely through the Internet when the Android smartphone was connected to cellular data. For example, a smartphone running any operating system could use a web browser application to display the temperature and humidity variation chart by browsing the user interface page of the Node-RED server.

**Figure 3:**
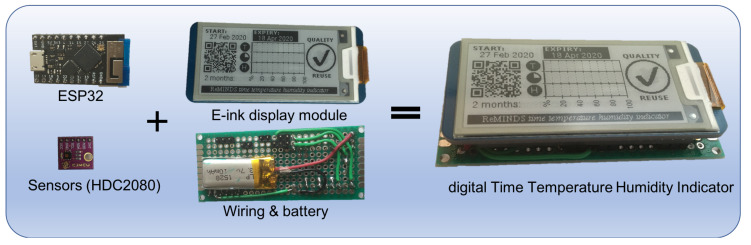
Hardware assembly of the dTTHI device.

The complete dTTHI device consists of a 2-in-1 sensor module (HDC2080), a ESP32 wireless SoC module, a 2.13 inch e-ink display module, and a 110 mAh rechargeable lithium battery assembled on a double sided prototyping Printed Circuit Board (PCB) (see Figure 3). The overall dimensions of the whole assembly was 80 mm (length) × 30 mm (width) × 16 mm (height) with the length and width governed by the chosen e-ink display. The height could be shrunk to a few millimetres when all necessary components were mounted on the back of the e-ink display module. However, the objective of current study was not to minimise the dimensions of a smart IoPT sensor; thus, only off-the-shelf components were used.

### 3.1. Firmware Implementation

**Figure 4:**
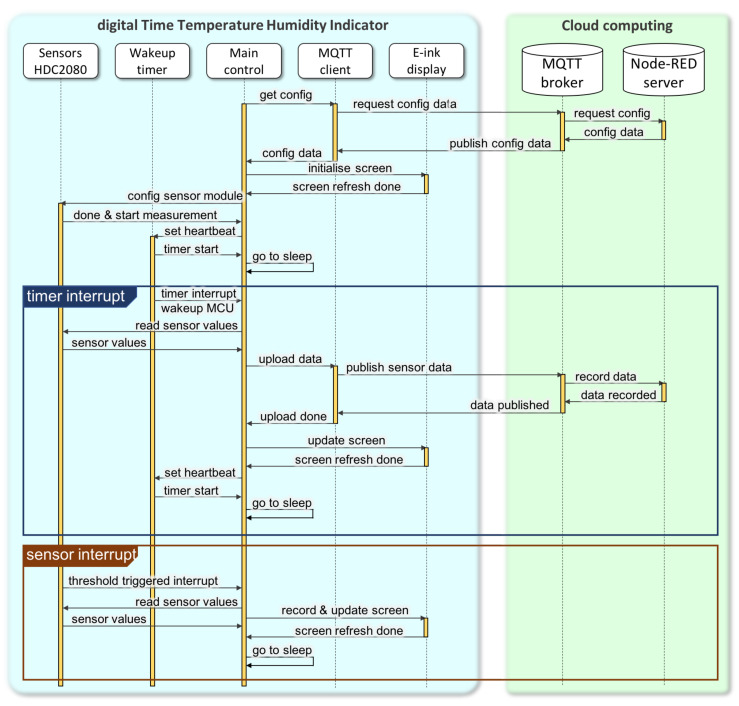
Sequence diagram of a dTTHI for medicine reuse based on the IoPT concept.

The dTTHI firmware was written using the Arduino IDE and its programming language (https://www.arduino.cc/en/Main/Software). Figure 4 illustrates a sequence diagram for the complete program including device management and interaction with cloud servers. Every dTTHI, we propose, will be assigned a unique identity code and the properties of the packaged medicine before it leaves the pharmaceutical company. An identity code enables the servers to know the configuration of the device for better communication with bespoke information, and this identity is linked to the corresponding medicine inside the package, such as the expiry date, and the high and low limits for environmental storage conditions, etc. Additionally, through the communication between servers and dTTHI devices, all these properties for packaged medicines can be updated if necessary (e.g., shorten the expiry date when the storage temperature exceeds predefined limits to an acceptable period of time, etc.). Once a packaged medicine is dispensed to a patient, the dTTHI is activated at the pharmacy and an initialisation starts.

**Figure 5:**
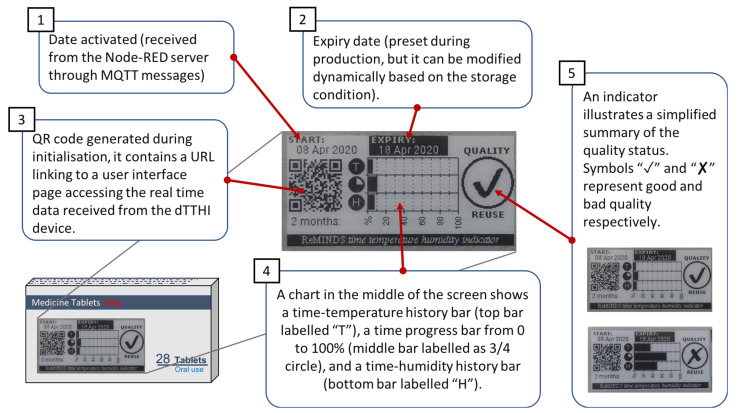
On-package display showing the real time quality status of the packaged medicine.

Initialisation of the dTTHI starts from registering the device identity code to the servers, and the current date and time as well as other updated information are pushed to the device (through MQTT publish/subscribe protocol). The current date and time information are important for a dTTHI device when power is reapplied the first time after leaving the pharmaceutical company. The e-ink display is refreshed illustrating the current status of the device (see Figure 5 for details of the screen content). The HDC2080 sensor module is also initialised to configure the automatic measuring frequency (suggested one measurement per minute), the high and low limits for both temperature and humidity readings to trigger a hardware interrupt to the main MCU, and also the mode of operation settings (e.g., taking both temperature and humidity for each measurement, enabling trigger pulse instead of trigger level, etc.). Finally, before the ESP32 module enters sleep mode, the wake-up timer is set to get the main MCU to work once per day. When the main MCU wakes up, it reads the sensor data, calculates the average value for all environmental parameters, updates the e-ink display, and turns on the radio to upload data to the cloud servers through MQTT messages. At the end of the initialisation session, the main MCU enters sleep mode and waits for interrupts from the sensor module or internal timer. The sensor module starts to measure temperature and humidity at a frequency set during initialisation, and its internal comparison logic triggers a hardware interrupt when the readings exceed the thresholds. The main MCU reads the sensor measurements after a hardware interrupt, stores the readings, updates the bar chart (i.e., how long the readings exceeded the thresholds), and refreshes the e-ink display content, but with the upload of data occurring at the wake-up interrupt scheduled by the timer.

The e-ink display enables local connectivity for patients or pharmacists. Five major components on the surface of pharmaceutical packaging, as shown in Figure 5 illustrate the real-time quality status of the packaged medicine: 1 shows the activation date (i.e., the date the medicine is dispensed at the pharmacy) of the dTTHI and is extracted from the Node-RED server during initialisation. 2 is the expiry date of the packaged medicine preset at the pharmaceutical company during production, however, it can be altered when necessary through the cloud servers (e.g., shorten the expiry date when the storage condition doesn’t meet the standard). 3 is a Quick Response (QR) code generated by the program during initialisation and it contains a link to access the user interface of the Node-RED server. A smartphone or any computing devices with a camera can scan the QR code and launch the web page automatically. This is a convenient way for the patients or other stakeholders to view the detailed information and sensing results of the dTTHI from the cloud servers. 4 shows a summary chart of the accumulated time-temperature and time-humidity history that the packaged medicine has experienced. The top bar in the chart labelled “**T**” is the accumulated time-temperature history, and the bottom bar labelled “**H**” represents the accumulated time-humidity history. The bar in the middle with a three-quarter circle label is the time bar which is a progress bar extending from 0% at the start and progresses to 100% at the end of the expiry date. Exceeding the temperature and/or humidity thresholds trigger the accumulation for the “**T**” and the “**H**” bars respectively. 5 is a simplified quality checker which shows a tick symbol if the quality of the packaged medicine is still in good condition for normal usage as well as for returning to the pharmacy for re-dispensing. On the other hand, if a cross symbol is displayed this depicts possible bad quality and the need for a decision on whether the medicine can be re-used to be taken by the pharmacists based on seeing the detail of the sensing data retrieved from the cloud servers.

**Figure 6:**
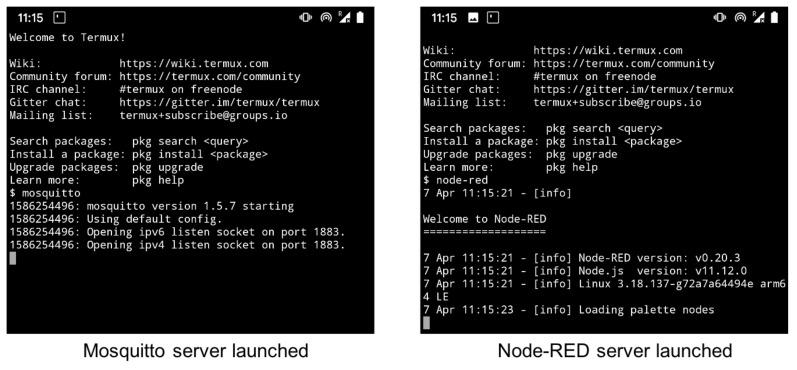
Screen capture of servers launched on Android smartphone.

For demonstration purposes, an Android smartphone was used to simulate a cloud computing environment. By using a simulated cloud computing platform this avoided the complicated free account setup process to commercial cloud services. This also provided a self-contained networking platform using an Android smartphone which enabled easy demonstration without an Internet connection. The smartphone was setup as a hotspot (or WiFi tethering), thus, it setup its own WiFi network. Three third party programs needed to be installed to setup the simulated cloud computing platform: Termux (https://termux.com), Mosquitto (https://mosquitto.org), and Node-RED (https://nodered.org/docs/getting-started/android). The Termux app was installed first in order to use the command line interface to install the other two programs, and Figure 6 illustrates the terminal screens for launching the two cloud server programs on an Android smartphone. For real life applications, these two servers as well as other cloud servers with different purposes would be hosted by internet service providers anywhere on the globe wherever an Internet connection is available.

## 4. Discussions

Developing an IoPT architecture using a dTTHI smart sensor as an example reveals the technological deficiency for building a complete ReMINDS medicine reuse ecosystem. Existing technologies provide ready-to-use tools and methodologies for building the IoPT conceptual framework with dTTHI as a functional component facilitating medicines returning and re-dispensing activities. Choosing off-the-shelf components could be one of the problems which mainly affects size and power consumption of the example smart sensor, but the lack of suitable technologies in many other functions may be the main obstacles.

A smart IoPT sensor that enhances pharmaceutical packaging to facilitate the reuse of medicines shares many characteristics of an IoT sensor node. Low power sensing and Internet connectivity apply to both IoPT and IoT sensors, but local connectivity between medicines and patients reveal major differences with regards to purpose and use. Technologies implemented on pharmaceutical packaging enable monitoring of the quality of packaged medicine and sharing of information remotely to every stakeholder through the Internet. Shaping the behaviour of patients in order to encourage them to return unused prescribed medicines requires local connectivity and is the first step in the medicine reuse concept. Providing an easy and convenient method for validating the quality and safety of medicines meets the requirements for stakeholders, especially patients and pharmacists, encouraging them to adopt a behavioural change and return to pharmacies unused medicines that are in good condition. The following sections depict the identified technological deficiencies from the present research through the development of the dTTHI smart sensor, and future research is proposed to overcome the observed obstacles according to the design requirements listed in Section 2.

### 4.1. Environmental Parameter Sensing

Sensor technologies for IoPT smart sensors may require seamless integration with packaging materials, thus, thin film electronics printable on papers could be a better choice. However, searching for sensor components during the design phase of the dTTHI has revealed that thin film sensors are not easy to find. Conversely, traditional silicon sensors with high sensing precision, ultra low power and small dimensions can be easily purchased off-the-shelf, and particularly the commonly used sensors for temperature, humidity and light intensity measurements. Other types of traditional silicon sensors for meeting the quality requirements listed in Section 2.1 such as agitation and contamination are not as common, but improvement of availability can be driven by demand. Thin film environmental sensors with digital interfaces that can be integrated onto packaging materials may be the most important research empowering the monitoring of storage conditions for the whole journey of the pharmaceutical supply chains. When re-dispensing of prescribed medicines is possible, all environmental parameters regarding storage conditions may need to be accumulated from multiple dispensing (and re-dispensing) paths between pharmacies and patients. Thus, the identification of sensing data for each distribution, as well as the accumulation of individual measurements are essential to establish a complete environmental sensing record.

Taking storage temperature monitoring in the UK as an example, there are standard operating procedures from the government or professional institutions restricting the storage conditions of medicines, where daily maximum and minimum readings are recorded for quality validation [31]. Different types of temperature sensitive medicines require different limitations on storage temperature variations (e.g., 2 °C to 8 °C for cold storage, 15 °C to 25 °C for room temperature storage, etc.). However, the sampling frequency for digital temperature measurements is not explicitly defined, so the accuracy of variation tracking may vary. Tracking of variation accuracy also leads to the definition of temperature range violation duration, which may affect the quality of packaged medicines. Mean Kinetic Temperature (MKT) may help evaluate the stability of medicines using the Arrhenius equation but the standards for MKT of individual pharmaceuticals are generally not available. Ref. Fu et al. [32] proposed to add humidity to the equation to extend quality validation with moisture variations. Additionally, standard acceptance levels for other environmental parameter violations are also required to facilitate a comprehensive quality validation for returned medicines after experiencing environmental stresses.

Legal issues restricting the re-dispensing of returned prescribed medicines may be related to the lack of quality and safety validation [9]. The recent temporary lifting of the restriction for reusing medicines in care homes and hospices in the UK due to the Covid-19 pandemic in 2020 [33], and the previous attempt to reuse expired medicines due to influenza pandemic in 2008 [34] reveal that returned medicines could be reused under national emergency. The NHS standard operating procedure allows pharmacists to reuse certain types of medicines according to their own judgement on the medicines quality. However, there is no tool helping them to perform the validation. IoPT smart sensors together with the architecture empowers a quality and safety validation based on empirical data which may help medicine reuse to become a common practice.

### 4.2. Expiry Date Monitoring and Management

A violation of expiry date overrides all other quality and safety validation results. A fixed expiry date written to a smart sensor associated with the packaged medicine before leaving the production line may not be good enough for changing situations. Degradation of the medication effectiveness due to storage condition violation may require a shortening of the expiry date, and a shortage of medicines due to national or international emergencies (such as the occurrence of a pandemic) may do the opposite and extend the expiry date. A proper procedure including the definition of parameters and chain of authorisation could be a possible research area proposing a dynamic expiry date management system for medicine reuse.

### 4.3. Safety Protection

Tamper monitoring sensors with digital interfaces help protect packaged medicines from contamination and/or adulteration [9]. Tamper-resistance and tamper-evident methodologies have been applied to pharmaceutical packaging [21]; however, detection of tampering through digital interfaces is not common. Anti-counterfeiting technologies provide another important safety protection to medicines and many overt and covert methodologies have been implemented in the packaging as well as in individual tablets. The latest candidate includes the emerging research using blockchain technology to register every process of the pharmaceutical supply chain into an unmodifiable online distributed ledger [35]. An addition of multiple re-dispensing processes may be a straightforward approach enabling medicine reuse as part of the blockchain protection algorithm.

### 4.4. Internet Connectivity

Internet connection is the core function in IoPT facilitating real-time information exchange among all stakeholders in the proposed ReMINDS ecosystem. The huge potential of the IoT market creates intense competition in wireless machines-to-machines communication protocols, and there is no single technology that can fit for all IoT applications [36]. WiFi, in spite of its comparatively high power consumption, has been a popular choice for connecting IoT devices to the Internet due to its pervasiveness and the ample support for development. The dTTHI smart sensor in the present research also uses WiFi for Internet connection, but the poor roaming performance reveals its deficiency in maintaining Internet connectivity along the whole supply chain. Further study in selecting the right technology and protocol is required to select the optimum configuration under the constraints of low installation and operation cost, low power consumption, and self-configurability.

### 4.5. Cloud Computing

Local and remote connectivity are both critical in the IoPT medicine reuse ecosystem. Remote connectivity linking medicines with all stakeholders in the pharmaceutical sector uses similar IoT technologies such as cloud computing. The dTTHI described in the last section utilised both MQTT and Node-RED servers to perform machines-to-machines and machines-to-humans communication respectively. Several dTTHIs and other smart sensors can connect to servers through the short message protocol with one or multiple MQTT servers, the Node-RED server can be used to act as an interface between users/stakeholders and other web services (e.g., artificial intelligent services, data analytics services, etc.). Cloud computing supporting the IoPT concept is relatively flexible such that various data analysis functions can be added dynamically once Internet connectivity is established.

### 4.6. Size and Power Consumption

Size and power consumption are common problems for IoT edge devices which require both to be as small as possible. Size may not be a very difficult problem to solve since the current silicon electronics can be minimised through nanotechnology. Regarding power consumption, the dTTHI is using a distributed processor architecture such that each individual feature (i.e., hardware) can be switched on and off to save power. An approximately half year battery life can be achieved using a single 110mAh lithium battery with a single charge. Further improvements can be made by lowering the standby current, which is currently rated at 25 uA and is much higher than the sensor operating current (500 nA). A custom made integrated circuit may help lower the standby current and be integrated with necessary features such as wireless radio connection. Energy harvesting may be a better solution but more research must be conducted for practical use.

### 4.7. Usability

Joining a network may not be trivial for computer novices, especially when a restricted user interface is provided. WiFi provisioning using a smartphone has been a common method where the network configuration settings (e.g., SSID, password, etc.) are transferred from the smartphone to the device needed to join the network. The existing dTTHI design also uses this method for provisioning which works well for users with computer knowledge. Configuring the WiFi router may be an alternative, but the need for basic networking knowledge could be an obstacle. LPWAN may provide a solution that does not require any user settings but it might incur a small connection fee. A public free (at least to the general users) long range network may be a solution in the future, and it may involve both technical and management research. This problem is correlated with the deficiency of Internet roaming discussed in Section 4.4. User provision for configuring an Internet connection will be eliminated once a seamless roaming is established.

Local connectivity plays a major role in enabling technology embedded pharmaceutical packaging to interact with patients. A human-centered design, as suggested by Norman [37], can communicate appropriate behaviours using proper signifiers (i.e., indicators). A high degree of usability may help motivate more medicine reuse activities since it could enhance the perceived control beliefs according to the TPB [8]. On-package indication such as the e-ink display used in the dTTHI provides a good example demonstrating a direct connection with patients (see Section 3.1 for details), where easy to understand indicators of the quality status of packaged medicine can be transferred to the patients and assist them to make the decision to return medicines. Using a smartphone or other computing device (block ⓖ in Figure 2) can offload the burden from the constrainted battery operated sensor nodes in terms of size and power consumption, but it will also degrade usability due to the additional operations. With proper screen content refresh cycle management (e.g., once per day in the dTTHI example), the overall energy consumption for a zero standby power e-ink display may not be significant compared to a wireless communication. A high resolution graphical display could have extra benefit for recycling the smart sensor since the screen content can be changed to reflect the different types of packaged medicines. An e-ink display can also be printed on thin film substrate but the cost of implementation is still on the high side. Further research on lower power and lower cost thin film graphical displays may be able to realise better alternatives.

### 4.8. Cost Effectiveness

Cost effectiveness analysis for a complete medicine reuse ecosystem based on the proposed IoPT concept is a complicated process which mainly involves (i) direct costs such as setup cost, running cost and product cost, (ii) indirect costs such as enhancing public health and reduce environmental pollution, and (iii) shared cost with pharmaceutical supply chain management such as Circular Economy (CE) [38]. Bekker et al. [39] review the pharmacy’s running cost and conclude that a re-dispensing of returned medicines is more effective for expensive medicines. Their findings illustrate that, at 10% return rate and 60% reuse rate, the costs for re-dispensing each medicine are EUR 101 (GBP 88.85) for room temperature storage, and EUR 215 (GBP 189.15) for cold storage. The costs drop to EUR 53 (GBP 46.63) and EUR 109 (GBP 95.89) when the return rate is improved to 10%. The higher the number of returned quality-validated medicines, the lower will be the re-dispensing cost. IoPT smart sensors may help improve the return rate through shaping patients’ behaviours, and also help increase the reuse rate through monitoring and validating quality and safety of returned medicines. A rough estimation of the product cost for building the dTTHI is GBP 10 based on the online small quantity (around 100 sets) purchase prices, however, the final product cost could be significantly lower when economy of scale is achieved. The setup cost is basically a one off expense and is not significant when basic cloud computing architecture is already established. The additional product cost on top of the running cost is also not significant for expensive medicines, however, the total direct cost per each re-dispensing of medicines is still high when the scope is expanded to every prescribed medicine. When there are 1 billion prescription items dispensed per year in the UK (2013 figure [3]), the cost for embedding IoPT smart sensors in every pharmaceutical packaging is huge. Recycling the smart sensors may help lower the per unit cost but extra management and administration is required.

Quantifying indirect cost effectiveness may not be straightforward, a cross-disciplinary research approach may be required to investigate the benefits gained from enhancing public health through better therapeutic effectiveness, enhancing medication adherence, and improving resources allocation. Environmental protection based on reducing improper medicinal waste disposal is also a benefit gain from medicine reuse, and a recent promotion from European governments provides better guidelines for sharing the cost with the CE through legislation, investment and production management (EU Action Plan for the Circular Economy [40]). Therefore, the cost of implementing a reuse scheme may be shared with the future CE transformation.

### 4.9. Incentive Facilitation

Providing incentives for stakeholders, especially patients and pharmacists, is not a complicated process. Registering each returning and re-dispensing transaction with hidden identity or through an anonymous process on a cloud server is enough to start a scheme involving every stakeholder. Credits or other benefits could be accumulated and associated with the successful reuse quantity. Cypersecurity must be implemented to protect privacy through available end-to-end security methodologies. However, as pointed out by Black [41], incentive schemes involving financial benefits may not be the best approach to motivate medicine reuse activities. Ensuring the integrity of medicines, enhancing public health and protecting the environment may be the aims of medicine reuse. More qualitative research should be conducted to investigate the best approach for setting an incentive scheme for medicine reuse.

### 4.10. Social Norms Support

Empirical and theoretical research has proved that human behaviours are affected by social norms [22]. Establishing a social networking platform is the minimum technological requirement and connecting Things to the IoPT will be the basic design criterion during development of the platform and its components. Motivating information sharing may be related to psychology and technologies may help translate the requirements into physical or virtual tools [42]. Artificial intelligent could be helpful in analysing human responses and provide visual aids to assist the shaping of stakeholders’ behaviours. Further multidisciplinary study on establishing a digital social norms network is required to understand the correct configuration for medicine reuse.

### 4.11. Security and Privacy Protection

Security and privacy protection is one of the major concerns to be resolved in the IoT. IoPT, as an extension of the IoT, shares all IoT technologies as well as all threats for potential malicious attacks. Distributed computing platforms including smart sensors (e.g., the dTTHI), cloud servers (e.g., the MQTT and NODE-Red servers), and different types of computing devices (e.g., devices in block ⓖ in Figure 2) are sharing resources to multiple applications in the ReMINDS ecosystem. The recent trend in IoT based Cyber Physical Systems (CPS) illustrates that connected devices are monitored and controlled by firmware, middleware and application software across multiple hardware platforms [43]. However, each individual platform can be independently developed and operated making data exchange capability, security and privacy protection more difficult to implement. Security and privacy considerations must be part of the design process from the beginning with security-by-design and privacy-by-design strategies adopted in every part of the whole IoPT architecture [44]. At the same time, individual security and privacy systems should be integrated to form one complete protection system for the whole IoPT architecture. End-to-end security protection must be implemented from individual smart sensors embedded in pharmaceutical packaging, to cloud servers holding all private and public stakeholders’ information. Hardware based security devices, such as cryptographic coprocessors [45], may be a solution to boost the security level of resource constrained edge devices like the dTTHI smart sensor. Embedded security hardware and firmware help improve protection from local physical attacks through secure boots, secure keys storage, and cryptographic acceleration, etc. If the addition of hardware secure elements is not feasible, offloading high processing power tasks, such as cryptography calculations, from resource constrained IoT edge devices can be done through edge or fog computing based on dedicated mechanisms [46,47]. A large amount of personal data including clinical reports, medication records, and personal identities is exchanged through the IoPT cloud servers where anonymous functions can help protect privacy to a certain extent [48], but merging with the emerging big data revolution for pharmaceuticals and healthcare systems is even more important [49,50]. Blockchain technology, again, may help enhance the security and privacy protection of detailed medicine reuse records in big data environments through registering all transactions in an unalterable distributed ledger [51]. Further research on finding a combination of technologies to implement appropriate security and privacy protection to the ReMINDS ecosystem is necessary.

### 4.12. Alternative Solutions

An alternative solution for implementing a complete ReMINDS ecosystem for reusing medicines is not yet available, but the applications of similar technologies for building part of the ecosystem are documented in a previous literature review [9]. smart (some previous research uses intelligent instead of smart) pill-boxes or medicine-boxes are emerging research which also study the applications of IoT technologies to medicines. They usually focus on enhancing medication adherence by embedding sensing and Internet connectivity to technology enabled boxes, and reminders are delivered wirelessly to patients through messaging or mobile computing devices [52,53,54]. The basic building blocks used in these studies are very similar to the present research, however, the main differences lie in the selection, integration and configuration of individual technologies. Environmental sensing for smart medicine-boxes is accomplished through embedded sensors on packaging or through a technology enabled box for housing packaged medicines, and the measurements normally focus on home storage environments. The dTTHI smart sensor monitors temperature and humidity variations along the whole pharmaceutical supply chain in order to capture any violation of standard storage conditions, and this record is critical for quality validation for re-dispensing returned medicines. The actions behind medicine reuse may be affected by human behaviours, thus, psychology also constitutes an important area of consideration during the ReMINDS ecosystem development.

### 4.13. Future Development Suggestions

Although local laws and regulations in the UK still prohibit the re-dispensing of returned prescribed medicines, the concept of medicine reuse is generally accepted by stakeholders of the pharmaceutical sector [9]. The benefit of reallocating these precious resources is further verified during national and international emergencies when legal restrictions are temporary lifted [55]. Quality and safety validation of returned medicines may be the main obstacles, where dedicated off-the-shelf tools can only be acquired occasionally to help verify their usable conditions. However, a complete self-sustaining system involving all stakeholders, medicines, validation tools, and management and administration systems should be established to facilitate the medicine reuse concept as a normal practice. The requirements for building the ReMINDS ecosystem are listed above and most of them require further research before they can be applied for development.

Table 2 lists the proposed research for each design requirement. The priority for conducting research activities is also stated in the table since some of the requirements are prerequisite for others, for examples, cost effectiveness consideration (high priority) should be defined before any hardware components are chosen, and security and privacy protection strategy (high priority) should be fixed before remote data communication is designed. Low priority status is reserved for those independent tasks that are less affected by other requirements, and all other tasks are assigned as medium priority. Suggestions on successfully using the requirements for further research and building the ecosystem are also illustrated in the table based on the popular SMART (Specific, Measurable, Attainable, Realistic and Timely) goal setting model [56]. Specifying clearly the outcomes of development are critical criteria in using the requirements for developing the ecosystem. Attention should also be paid to any conflicts between requirements, for example, having high usability may degrade the attainable level of security, and the frequency of sensor measurements is inversely proportional to power saving. Table 3 depicts the relationship between each requirement discussed above.

## 5. Conclusions

This paper presents an IoPT concept for building the ReMINDS ecosystem, which was proposed by the same authors to facilitate medicine reuse [9]. As an IoPT smart sensor, a dTTHI was built as an example illustrating an application of the design concept derived from the ReMINDS ecosystem. Basic design requirements were translated from the IoPT concept, and were used to select the components, to define the hardware architecture, to determine the sequence of operations, and to design the firmware. Specific attention was paid to local connectivity where stakeholders, especially patients, could use the information received from the on-package display to take actions through a perceived behavioural control. Existing technologies can be used to build the dTTHI but they cannot meet all IoPT design requirements. Design restrictions such as size and power consumption may need to be enhanced. Thin film printed electronics can be used to integrate digital electronics onto the packaging materials such as paper but the technology is not yet mature enough. A 110 mAh lithium battery can keep the dTTHI working for almost half year but a reasonable target may need to be extended to a few years, with energy harvesting as a potential solution. Supporting functions such as cloud computing, usability, cost effectiveness, incentive facilitation, social norms support, and security and privacy protection require more research to build a complete medicine reuse ecosystem. 

## Figures and Tables

**Table 1:** Major requirements for medicine reuse through qualitative research (data extracted from Hui et al. [9]).

**Qualitative Research**	Patients’ perspective [7] Healthcare professionals’ perspective [5] Stakeholders’ perspective [6] Theory of Planned Behaviour (TPB) [8]
**Quality Requirements**	Storage temperature monitoring Storage humidity monitoring Storage lighting monitoring Storage contamination monitoring Agitation monitoring Lapsed expiration date monitoring
**Safety Requirements**	Tamper-proof packaging Anti-counterfeit Track and trace collecting and dispensing system Errors tracking from patients and pharmacists
**Other Requirements**	Patients’ incentive for returning unwanted medicines Pharmacists’ incentive for extra workload in re-dispensing medicines Cost effectiveness monitoring of reusing medicines Legal issues such as legislation on re-dispensing medicines and professional standards for pharmacists Social norms for promoting medicine reuse On-site and off-site collection and distribution system

**Table 2:** Proposed research for developing the ReMINDS ecosystem.

Proposed Development Requirements and Suggested Future Research	SMART Goal Setting Suggestions(*S:* Specific, *M:* Measurable, *A:* Attainable, *R:* Realistic, *T:* Timely)
Environmental parameter sensing: study and propose thin-film environmental sensors for quality validation (see Section 2.1 for the required sensors); research on optimum sensing configurations (frequency, high and low limits, etc.); research on quality validation standards (e.g., MKT, violation ranges, etc. ). Priority = medium	***S:*** extract validation guidelines from existing standard operating procedures from medical professionals, government and pharmaceutical companies. ***M:*** separate each sensor design into controllable phases. ***A:*** only existing technologies are chosen. ***R:*** using existing standards from pharmaceutical companies for every type of medicines as sensing target. ***T:*** divide whole project into several phases to produce timely outcomes.
Expiry date monitoring and management: study and propose a dynamic expiry date management system for medicine reuse. Priority = medium	***S:*** define a dynamic expiry date system with remote access through Internet connectivity, and the rules to change can be configurable at any time after the system is complete. ***M:*** a clearly defined design specification can be used as validation tool. ***A:*** using existing IoT technologies. ***R:*** using existing IoT technologies. ***T:*** divide whole project into several phases to produce timely outcomes.
Safety protection: research on building tamper monitoring sensors with digital interfaces; research on choosing suitable counterfeit protection algorithm targeted for medicines (e.g., applying blockchain technology for pharmaceutical supply chains, etc.). Priority = medium	***S:*** for tampering, need to define the types of tampering for study to limit the scope since there are too many tampering methods being used; for anti-counterfeit, try to expand the existing technology to include medicines as part of the system. ***M:*** separate goal into achievable objectives. ***A:*** based on application of existing technologies. ***R:*** based on application of existing technologies. ***T:*** divide whole project into several phases to produce timely outcomes.
Internet connectivity: study, compare and propose a low power, low cost Internet connectivity with automatic and seamless roaming for building the ReMINDS ecosystem (e.g., WiFi, LPWAN, cellular, etc.). Priority = medium	***S:*** choose existing technology according to specific requirements (automatic roaming, low cost, low power, etc.). ***M:*** using existing IoT technologies. ***A:*** using existing IoT technologies. ***R:*** using existing IoT technologies. ***T:*** divide whole project into several phases to produce timely outcomes.
Cloud computing: study and propose a cloud computing platform for both machines to machines and machines to human communications, focusing on extensible and reconfigurable for future upgrade. Priority = low	***S:*** choose existing cloud computing technology according to specific requirements (extensible and reconfigurable cloud computing platform). ***M:*** using existing IoT technologies. ***A:*** using existing IoT technologies. ***R:*** using existing IoT technologies. ***T:*** divide whole project into several phases to produce timely outcomes.
Size and power consumption: study and choose the correct thin-film technology applicable to pharmaceutical packaging; research and select suitable components with low standby power; study and propose a power management method to minimise overall power consumption. Priority = high	***S:*** need to first define the maximum size and power budget before conducting any study, may provide several class of solutions to fit for different medicinal products if no single architecture can resolve all concerns. ***M:*** separate goal into achievable objectives. ***A:*** based on application of existing technologies. ***R:*** based on application of existing technologies. ***T:*** divide whole project into several phases to produce timely outcomes.
Usability: study and define user interfaces based on user experience study; research and suggest low cost and low power indicators (e.g., e-ink or electrochromic display printed on packaging materials, etc.); qualitative research on user interface design to facilitate or motivate return of unused medicines. Priority = medium	***S:*** user survey is critical in defining the minimum requirements for user interfaces that facilitate medicine reuse through shaping patients’ behaviours, it also affects the form factor of indications and the context to be delivered to stakeholders. ***M:*** separate goal into achievable objectives. ***A:*** based on application of existing technologies. ***R:*** based on application of existing technologies. ***T:*** divide whole project into several phases to produce timely outcomes.
Cost effectiveness: study and estimate total cost to implement the ReMINDS ecosystem with consideration of direct, indirect and shared costs (see Section 4.8 for details); define cost budget as a guideline for cost analysis in terms of scale and scope of implementation; setup cost control for each design requirements from the cost budget as one of the development guidelines. Priority = high	***S:*** a detail study is conducted initially to define the way for sharing the project cost, and a reasonable estimation of annual saving is proposed as the baseline to derive the individual budgets for each sub-system. This exercise is the first step in building the ReMINDS ecosystem based on the IoPT concept, thus, it is of the highest priority. the highest ***M:*** estimation based on empirical data from previous research and current quantitative study from the pharmaceutical sector. ***A:*** based on application of existing technologies. ***R:*** based on application of existing technologies. ***T:*** divide whole project into several phases to produce timely outcomes.
Incentive facilitation: qualitative research on incentive scheme through collecting data from all stakeholders of the pharmaceutical sector; study and propose an implementation of the incentive scheme on the defined cloud computing platform. Priority = low	***S:*** conduct cross-disciplinary qualitative research to define what incentive schemes are acceptable to the stakeholders and define the criterion for implementing the scheme on cloud computing. ***M:*** using existing IoT technologies. ***A:*** using existing IoT technologies. ***R:*** using existing IoT technologies. ***T:*** divide whole project into several phases to produce timely outcomes.
Social norms support: qualitative research on the requirements and configurations of social norms through collecting data from all stakeholders of the pharmaceutical sector; study and propose an implementation of a digital social norms network on the defined cloud computing platform. Priority = low	***S:*** conduct cross-disciplinary qualitative research to define what social norms settings are appropriate to motivate stakeholders’ behaviours and define the criterion for implementing the digital social norms network on cloud computing. ***M:*** using existing IoT technologies. ***A:*** using existing IoT technologies. ***R:*** using existing IoT technologies. ***T:*** divide whole project into several phases to produce timely outcomes.
Security and privacy protection: research and define the security and privacy protection strategy for the whole ReMINDS ecosystem; setup design guidelines for individual components from IoPT edge devices to cloud servers. Priority = high	***S:*** try to expand the existing technology, especially big data for healthcare and blockchain technologies, to include medicines as part of the security and privacy protection system. ***M:*** separate goal into achievable objectives. ***A:*** based on application of existing technologies. ***R:*** based on application of existing technologies. ***T:*** divide whole project into several phases to produce timely outcomes.

**Table 3:** Conflicts between proposed design requirements for developing the ReMINDS ecosystem (a “X” symbol illustrates a conflict between the column and row requirements).

Conflicts Between Design Requirements	Environmental Parameter Sensing	Expiry Date Monitoring and Management	Safety Protection	Internet Connectivity	Cloud Computing	Size and Power Consumption	Usability	Cost Effectiveness	Incentive Facilitation	Social Norms Support	Security and Privacy Protection
Environmental parameter sensing						X		X			
Expiry date monitoring and management						X		X			
Safety protection						X		X			
Internet connectivity						X		X			X
Cloud computing											X
Size and power consumption	X	X	X	X			X	X			
Usability						X		X			X
Cost effectiveness	X	X	X	X		X	X				X
Incentive facilitation											X
Social norms support											X
Security and privacy protection				X	X		X	X	X	X	

## References

[1] Trueman P., Taylor D., Lowson K., Bligh A., Meszaros A., Wright D., Glanville J., Newbould J., Bury M., Barber N. (2010). Evaluation of the Scale, Causes and Costs of Waste Medicines, Technical Report, DH Funded National Project. https://discovery.ucl.ac.uk/id/eprint/1350234/1/Evaluation_of_NHS_Medicines_Waste__web_publication_version.pdf.

[2] Singleton J.A., Nissen L.M., Barter N., McIntosh M. (2014). The global public health issue of pharmaceutical waste: What role for pharmacists?. J. Glob. Responsib..

[3] Hazell B., Robson R. Pharmaceutical Waste Reduction in the NHS. https://www.england.nhs.uk/wp-content/uploads/2015/06/pharmaceutical-waste-reduction.pdf.

[4] Connelly D. (2019). Should pharmacists be allowed to reuse medicines. Pharm. J..

[5] McRae D., Allman M., James D. (2016). The redistribution of medicines: Could it become a reality?. Int. J. Pharm. Pract..

[6] Bekker C.L., Gardarsdottir H., Egberts T.C.G., Bouvy M.L., van den Bemt B.J.F. (2017). Redispensing of medicines unused by patients: A qualitative study among stakeholders. Int. J. Clin. Pharm..

[7] Bekker C., van den Bemt B., Egberts T.C.G., Bouvy M., Gardarsdottir H. (2019). Willingness of patients to use unused medication returned to the pharmacy by another patient: A cross-sectional survey. BMJ Open.

[8] Alhamad H., Patel N., Donyai P. (2018). How do people conceptualise the reuse of medicines? An interview study. Int. J. Pharm. Pract..

[9] Hui T.K.L., Mohammed B., Donyai P., McCrindle R., Sherratt R.S. (2020). Enhancing pharmaceutical packaging through a technology ecosystem to facilitate the reuse of medicines and reduce medicinal waste. Pharmacy.

[10] Kuswandi B., Wicaksono Y., Jayus Abdullah A., Lee Y.H., Ahmad M. (2011). Smart packaging: Sensors for monitoring of food quality and safety. Sens. Instrum. Food Qual. Saf..

[11] Sehgal S., Jaithliya T., Khan M., Devi A.N., Banoo J., Tiwari A. (2018). Recent trends and future of pharmaceutical packaging technology: An overview. Eur. J. Biomed. Pharm. Sci..

[12] Christensson P. SMART (Self-Monitoring Analysis And Reporting Technology) Definition. https://techterms.com/definition/smart.

[13] Lorenzini G.C., Mostaghel R., Hellstrom D. (2018). Drivers of pharmaceutical packaging innovation: A customer-supplier relationship case study. J. Bus. Res..

[14] Wang S., Liu X., Yang M., Zhang Y., Xiang K., Tang R. (2015). Review of time temperature indicators as quality monitors in food packaging. Packag. Technol. Sci..

[15] Mijanur Rahman A.T.M., Kim D.H., Jang H.D., Yang J.H., Lee S.J. (2018). Preliminary study on biosensor-type time-temperature integrator for intelligent food packaging. Sensors.

[16] Zhang X., Shan X., Wei J. Hybrid Flexible Smart Temperature Tag with NFC Technology for Smart Packaging. Proceedings of the IEEE 19th Electronics Packaging Technology Conference.

[17] Madhusudan P., Chellukuri N., Shivakumar N. (2018). Smart packaging of food for the 21st century—A review with futuristic trends, their feasibility and economics. Mater. Today Proc..

[18] Kuswandi B., Moradi M. (2019). Sensor trends in beverages packaging. Trends Beverage Packag..

[19] Johnston J.J., Wong J.P., Feldman S.E., Ilnicki L.P. (1994). Purge and trap/gas chromatography/mass spectrometry method for determining smoke contamination of foods and packaging materials. J. Agric. Food Chem..

[20] Colberg L., Schmidt-Petersen L., Hansen M.K., Larsen B.S., Otnes S. (2017). Incorrect storage of medicines and potential for cost savings. Eur. J. Hosp. Pharm..

[21] Kumar A.K., Gupta N.V., Lalasa P., Sandhil S. (2013). A review on packaging materials with anti-counterfeit, tamper-evident features for pharmaceuticals. Int. J. Drug Dev. Res..

[22] Yamin P., Fei M., Lahlou S., Levy S. (2019). Using social norms to change behavior and increase sustainability in the real world: A systematic review of the literature. Sustainability.

[23] Maity M., Bagchi K., Shah A., Misra A. (2019). Explaining normative behavior in information technology use. Inf. Technol. People.

[24] Piyare R., Murphy A.L., Kiraly C., Tosato P., Brunelli D. (2017). Ultra low power wake-up radios: A hardware and networking survey. IEEE Commun. Surv. Tutor..

[25] Jang T., Choi M., Shi Y., Lee I., Sylvester D., Blaauw D. Millimeter-Scale Computing Platform for Next Generation of Internet of Things. Proceedings of the RFID 2016: International IEEE Conference on RFID.

[26] Hui T.K.L., Sherratt R.S. (2017). Towards disappearing user interfaces for ubiquitous computing: Human enhancement from sixth sense to super senses. J. Ambient. Intell. Humaniz. Comput..

[27] Koomey J., Naffziger S. (2015). Moore’s Law might be slowing down, but not energy efficiency. IEEE spectrum.

[28] Texas Instruments Low-Power Humidity and Temperature Digital Sensor. http://www.ti.com/lit/ds/symlink/hdc2080.pdf.

[29] Espressif Systems ESP32 Series Datasheet. https://www.espressif.com/sites/default/files/documentation/esp32_datasheet_en.pdf..

[30] Waveshare Electronics (2017). Specification EPD Screen Size: 2.13", Color: Black and White, Display Resolution: 250*122. https://www.waveshare.com/w/upload/e/e6/2.13inch_e-Paper_Datasheet.pdf..

[31] Taylor J. (2001). Recommendations on the control and monitoring of storage and transportation temperatures of medicinal products. Pharm. J..

[32] Fu M., Perlman M., Lu Q., Varga C. (2015). Pharmaceutical solid-state kinetic stability investigation by using moisture-modified Arrhenius equation and JMP statistical software. J. Pharm. Biomed. Anal..

[33] NHS England (2020). Novel Coronavirus (COVID-19) Standard Operating Procedure: Running a Medicines Re-Use Scheme in a Care Home or Hospice Setting. https://assets.publishing.service.gov.uk/government/uploads/system/uploads/attachment_data/file/881838/medicines-reuse-in-care-homes.pd.

[34] Boyd M. Council Approves Use of Patient-Returned and Date-Expired Medicines in the Event of Pandemic Flu. https://www.pharmaceutical-journal.com/news-and-analysis/council-approves-use-of-patient-returned-and-date-expired-medicines-in-the-event-of-pandemic-flu/10036098.article.

[35] Nørfeldt L., Bøtker J., Edinger M., Genina N., Rantanen J. (2019). Cryptopharmaceuticals: Increasing the safety of medication by a blockchain of pharmaceutical products. J. Pharm. Sci..

[36] Andreev S., Galinina O., Pyattaev A., Gerasimenko M., Tirronen T., Torsner J., Sachs J., Dohler M., Koucheryavy Y. (2015). Understanding the IoT connectivity landscape: A contemporary M2M radio technology roadmap. IEEE Commun. Mag..

[37] Norman D. (2013). The Design of Everyday Things: Revised and Expanded Edition. Basic Books. https://www.basicbooks.com/titles/don-norman/the-design-of-everyday-things/9780465050659/.

[38] Viegas C.V., Bond A., Vaz C.R., Bertolo R.J. (2019). Reverse flows within the pharmaceutical supply chain: A classificatory review from the perspective of end-of-use and end-of-life medicines. J. Clean. Prod..

[39] Bekker C.L., Gardarsdottir H., Egberts A.C.G., Molenaar H.A., Bouvy M.L., van den Bemt B.J.F., Hövels A.M. (2019). What does it cost to redispense unused medications in the pharmacy? A micro-costing study. Bmc Health Serv. Res..

[40] European Commission (2020). Circular Economy Action Plan: For a Cleaner and More Competitive Europe. Report..

[41] Black G. (2011). Reuse of medicine: It’s not about the money!: From my Little Black Book of pharmacy practice: Practice matters. Pharm. J..

[42] Spottswood E.L., Hancock J.T. (2017). Should I share that? Prompting social norms that influence privacy behaviors on a social networking site. J. Comput. Mediat. Commun..

[43] Karsai G., Balasubramanian D., Dubey A., Otte W.R. Distributed and Managed: Research Challenges and Opportunities of the Next Generation Cyber-Physical Systems. Proceedings of the 17th IEEE International Symposium on Object/Component/Service-Oriented Real-Time Distributed Computing (ISORC).

[44] Geismann J., Gerking C., Bodden E. Towards Ensuring Security by Design in Cyber-Physical Systems Engineering Processes. Proceedings of the International Conference on Software and System Process (ICSSP).

[45] Rashid M., Imran M., Jafri A.R. Comparative Analysis of Fexible Cryptographic Implementations. Proceedings of the 11th International Symposium on Reconfigurable Communication-Centric Systems-on-Chip (ReCoSoC).

[46] Wang J., Wu W., Liao Z., Sherratt R.S., Kim G., Alfarraj O., Alzubi A., Tolba A. (2020). A probability preferred priori offloading mechanism in mobile edge computing. IEEE Access.

[47] Farahani B., Firouzi F., Chang V., Badaroglu M., Constant N., Mankodiya K. (2018). Towards fog-driven IoT eHealth: Promises and challenges of IoT in medicine and healthcare. Future Gener. Comput. Syst..

[48] Yin X.C., Liu Z.G., Ndibanje B., Nkenyereye L., Riazul Islam S. (2019). An IoT-based anonymous function for security and privacy in healthcare sensor networks. Sensors.

[49] Wang J., Yang Y., Wang T., Sherratt R.S., Zhang J. (2020). Big Data Service Architecture: A Survey. J. Internet Technol..

[50] Abouelmehdi K., Beni-Hessane A., Khaloufi H. (2018). Big healthcare data: Preserving security and privacy. J. Big Data.

[51] Dwivedi A.D., Srivastava G., Dhar S., Singh R. (2019). A decentralized privacy-preserving healthcare blockchain for IoT. Sensors.

[52] Yang G., Xie L., Mantysalo M., Zhou X.L., Pang Z.B., Xu L.D., Kao-Walter S., Chen Q., Zheng L.R. (2014). A health-IoT platform based on the integration of intelligent packaging, unobtrusive bio-sensor, and intelligent medicine box. IEEE Trans. Ind. Informatics.

[53] Tsai H., Tseng C.H., Wang L., Juang F. Bidirectional Smart Pill Box Monitored through Internet and Receiving Reminding Message from Remote Relatives. Proceedings of the IEEE International Conference on Consumer Electronics-Taiwan (ICCE-TW).

[54] Abdul Minaam D.S., Abd-Elfattah M. (2018). Smart drugs: Improving healthcare using smart pill box for medicine reminder and monitoring system. Future Comput. Inform. J..

[55] Donyai P., McCrindle R., Sherratt R.S., Hui T.K.L. COVID-19 Pandemic Is Our Chance to Learn How to Reuse Old Medicines. https://theconversation.com/covid-19-pandemic-is-our-chance-to-learn-how-to-reuse-old-medicines-137671?utm_source=twitter&utm_medium=bylinetwitterbutton..

[56] Bjerke M.B., Renger R. (2017). Being smart about writing SMART objectives. Eval. Prog. Plan..

